# Kinetics of Serologic Responses to MERS Coronavirus Infection in Humans, South Korea

**DOI:** 10.3201/eid2112.151421

**Published:** 2015-12

**Authors:** Wan Beom Park, Ranawaka A.P.M. Perera, Pyoeng Gyun Choe, Eric H.Y. Lau, Seong Jin Choi, June Young Chun, Hong Sang Oh, Kyoung-Ho Song, Ji Hwan Bang, Eu Suk Kim, Hong Bin Kim, Sang Won Park, Nam Joong Kim, Leo Lit Man Poon, Malik Peiris, Myoung-don Oh

**Affiliations:** Seoul National University College of Medicine, Seoul, South Korea (W.B. Park, P.G. Choe, S.J. Choi, J.Y. Chun, H.S. Oh, K.-H. Song, J.H. Bang, E.S. Kim, H.B. Kim, S.W. Park, N.J. Kim, M.-d. Oh);; The University of Hong Kong, Pokfulam, Hong Kong, China (R.A.P.M. Perera, E.H.Y. Lau, L.L.M. Poon);; Hong Kong University–Pasteur Research Pole, Pokfulam (M. Peiris)

**Keywords:** Middle East respiratory syndrome coronavirus, MERS-CoV, Middle East respiratory syndrome, MERS, coronavirus, antibody, kinetics, immunity, severity, viruses, South Korea, clinical correlates, serologic responses, diagnostics, seroepidemiologic data, humans

## Abstract

We investigated the kinetics of serologic responses to Middle East respiratory syndrome coronavirus (MERS-CoV) infection by using virus neutralization and MERS-CoV S1 IgG ELISA tests. In most patients, robust antibody responses developed by the third week of illness. Delayed antibody responses with the neutralization test were associated with more severe disease.

Knowledge of the kinetics and clinical correlates of serologic responses to Middle East respiratory syndrome coronavirus (MERS-CoV) infection is essential for diagnosing the disease, interpreting seroepidemiologic data to define prevalence and risk factors for infection, understanding pathogenesis, and assessing a potential role for passive immunotherapy. To address this knowledge gap, we investigated serologic responses to MERS-CoV in 17 patients.

## The Study

During May–June 2015, an outbreak of MERS-CoV in South Korea resulted in 186 infections and 36 deaths (*1*–*3*); the outbreak strain was a clade B MERS-CoV closely related to viruses circulating in the Middle East (*1*). Seventeen patients with reverse transcription PCR–confirmed MERS-CoV infections were included in this study; the patients were hospitalized at Seoul National University (SNU) Hospital or SNU Boramae Medical Center in Seoul, South Korea, or at SNU Bundang Hospital, in Bundang, South Korea. We investigated early serologic responses; thus, patients who were transferred to these facilities >14 days after illness onset were excluded from study. Patients’ demographic and clinical profiles are shown in Technical Appendix Table 1. Of the 17 patients, 9 had severe disease (4 required mechanical ventilation, 4 required supplemental oxygen; 1 died) and 8 had mild disease. Serial serum samples were collected and analyzed. The study was approved by the SNU Institutional Review Board.

Antibody to MERS-CoV was detected by using the plaque reduction neutralization test (PRNT) and MERS-CoV S1 IgG ELISA (EUROIMMUN, Lübeck, Germany) (*4*,*5*) (Technical Appendix). MERS-CoV EMC was used for the PRNT assay; a 50% PRNT endpoint (PRNT_50_) was used because it was more sensitive than the 90% PRNT cutoff in detecting mild infections (*6*). The ELISA was based on the recombinant spike S1 region of strain EMC because that region is sufficiently divergent between different coronavirus species and expected to lead to less cross-reaction (*4*).

Overall, serologic responses were robust and were detected in most patients by week 3 of illness (Figure). Of the 12 patients who had serum samples tested beyond day 18 of illness, 9 had PRNT_50_ titers of 1:320 by day 21 and 2 more had titers >1:320 by day 28. Patient L, a 56-year-old woman with no underlying disease, had weakly positive PRNT_50_ (1:20) and borderline ELISA responses (optical density ratio 1.0), even at day 32 of illness. A chest radiograph showed she had lung infiltrates, but she was not oxygen-dependent and was not administered antiviral drugs or corticosteroids; her recovery was uneventful.

**Figure:**
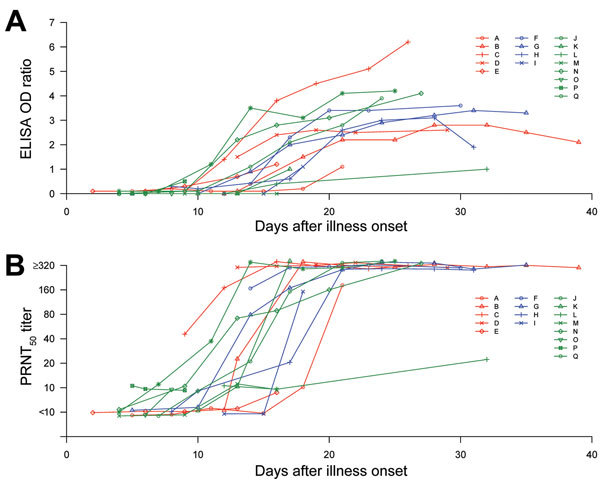
Antibody response kinetics in patients with Middle East respiratory syndrome coronavirus (MERS-CoV) infection, by days after illness onset, as determined by using a 50% endpoint plaque reduction neutralization test (PRNT_50_) (A) and an S1 IgG ELISA (B). Key indicates individual patients; Red indicates patients with severe illness requiring mechanical ventilation; blue indicates patients with severe illness requiring only supplemental oxygen therapy; and green indicates patients with mild illness. For better presentation, the PRNT_50_ titers have been jittered vertically (random noise added to prevent overplotting) (*7*) by adding random numbers to the titers within the range of −0.2 to 0.2 at the log scale. OD, optical density.

Antibody responses in patient A, a 38-year-old man, were delayed up to 16–18 days after illness onset (Figure). He required mechanical ventilation, and on illness day 14, he was given convalescent-phase plasma (200 mL; antibody titer unknown) from the outbreak index patient’s wife (*1*). The next day, antibody responses were undetectable in the patient’s serum by PRNT or ELISA. By day 18, he had a PRNT_50_ antibody titer of 1:10 and a negative ELISA response; strong antibody responses developed from day 21 onwards. We hypothesize that the data from the first 21 days of illness represent his own serologic response, unaffected by the passive transfusion with convalescent-phase plasma on day 14; thus, these data were included in the analysis. Patient A was given a second infusion of convalescent-phase plasma on day 24, and serologic data after day 21 were excluded from analysis.

We constructed a statistical model in which age, sex, incubation period, concomitant conditions, and therapy with corticosteroids or antiviral drugs were adjusted for disease severity. We assessed how these factors were associated with the time from illness onset to commencement of the log-phase antibody response (Table 1) and the time for the antibody response to reach a titer of 1:40 (PRNT_50_) or become positive in the ELISA (Technical Appendix Table 2). An accelerated failure model was used for a more natural interpretation of the median time from illness onset to the aforementioned antibody responses (Technical Appendix). Because the increase in antibody titers exhibited an S-shaped pattern, we assessed the rate of change in antibody response after the commencement of the exponential phase by manually removing data from the steady state, thus restricting antibody data to the log-phase response (Table 2). A linear mixed model was used to test the potential difference in the rate of increase by the above factors (Technical Appendix). Patients with severe disease had significant delays in the commencement of PRNT_50_ antibody responses (Table 1) but had a steeper slope to the antibody response once it began (Table 2). Thus, a delayed adaptive immune response may contribute to increased severity, and passive therapy with convalescent-phase immune plasma may be clinically beneficial. In avian influenza A(H7N9) virus infection of humans, earlier antibody responses and a faster rate of increasing antibody titers were associated with milder disease (*8*), but in SARS-CoV infection, earlier antibody responses were associated with an adverse outcome (*9*).

**Table 1:** Associations and p values for different clinical factors with time from illness onset to commencement of log phase of antibody response in PRNT_50_ and S1-ELISA*

Clinical factors	Acceleration factor of time from illness onset to log phase of antibody response				
	PRNT_50_ titer	p value		S1-ELISA OD ratio‡	p value
Severe disease	1.61	<0.001		1.19	0.21
Male sex†	0.90	0.52		0.90	0.48
Age >60 y†	0.95	0.73		1.08	0.60
Incubation period, d†	0.97	0.06		0.95	<0.001
Use of corticosteroid†	1.19	0.33		1.14	0.47
Use of antiviral drugs†	1.07	0.61		0.76	0.03
Concomitant conditions†	1.08	0.57		1.15	0.30

*Accelerated failure time models were used; acceleration factor >1 means a longer interval to commencement of antibody response. OD, optical density; PRNT_50_, 50% endpoint plaque reduction neutralization test. †Effects were adjusted for severity. ‡Increase over S1-ELISA OD >0.8..

**Table 2:** Testing potential difference in rates of change in antibody titers over day of illness during the exponential phase of the antibody response, accounting for sequential measurements taken at different days of illness and adjusted for severity*

Clinical factors	Difference in rates of change in log antibody titers				
	PRNT_50_ titer	p value		S1-ELISA OD ratio	p value
Severe disease	0.09	0.01		0.08	0.07
Male sex†	0.07	0.05		0.14	0.01
Age >60 y†	0.05	0.22		−0.03	0.65
Incubation period, d†	0.01	0.16		0.02	0.004
Use of corticosteroid†	0.06	0.37		−0.04	0.58
Use of antiviral drugs†	0.06	0.10		0.05	0.35
Concomitant conditions†	0.06	0.06		0.07	0.16

*Differences in rates of change and p values were estimated by using linear mixed models; positive value indicates a faster increase in antibody titer. Given that the antibody titers exhibited an S-shaped pattern, the analysis was restricted to data for log-phase antibody responses by manually removing data from the inductive/steady-state phase. Increases in antibody titers during the log phase were compared by different factors, adjusted for disease severity, by using a linear mixed model to account for repeated measurements, assuming a linear increasing trend by days since illness onset. PRNT_50_ titers were first log-transformed (with base 10). OD, optical density; PRNT_50_, 50% endpoint plaque reduction neutralization test. †Effects were adjusted for severity.

Extensive contact tracing during the outbreak enabled us to determine the date of MERS-CoV exposure and incubation periods for patients (Technical Appendix Table 1). A longer incubation period was associated with earlier commencement of antibody responses detectable by ELISA (Table 1; Technical Appendix Table 2) and with a steeper slope to the response once it began (Table 2). Even after adjusting for disease severity, the use of interferon and antiviral drugs was associated with earlier commencement of antibody responses detectable by ELISA (Table 1). The time to commencement of response was similar for men and women, but the slope of the response was steeper for male patients (Table 2).

## Conclusions

An understanding of MERS-CoV antibody response kinetics helps in defining the window during which passive antibody therapy may be useful. In our study, this window was the first 21 days of illness for most patients. However, some patients may not develop strong antibody responses even after 4 weeks of illness, so therapy must be individualized.

Our study has some limitations. First, no MERS-CoV isolates from the study patients were available, so MERS-CoV EMC was the basis of the serologic assays we used. Strain EMC is a clade A virus, and the outbreak in South Korea was caused by a clade B virus (*1*). However, using serum from naturally infected camels, we previously showed that clade A and B viruses and genetically diverse MERS-CoVs from Egypt were serologically indistinguishable (*10*). Another study reported that isolates of MERS-CoVs circulating in Saudi Arabia in 2014 were antigenically indistinguishable from the EMC strain in neutralization tests with human convalescent-phase serum (*5*). Thus, it is unlikely that the use of MERS-CoV EMC in our study considerably affected the observed antibody titers. A second limitation was the small number of patients studied (n = 17) and that they were followed only through the acute stage of illness. Longer term follow-up is needed to define the duration of antibody responses. If MERS-CoV antibody responses wane, as has been reported with SARS (11), this is relevant for interpretation of seroepidemiologic studies and for finding convalescent-phase donors with high antibody titers for passive immunotherapy. It would be useful to investigate IgM antibody responses and antibody responses to other virus proteins, including the MERS-CoV nucleoprotein, especially in patient L, who had poor antibody responses.

In summary, our findings showed that an early MERS-CoV antibody response was associated with reduced disease severity. Robust neutralizing and S1 ELISA IgG antibody responses were mounted by the third week of illness in most patients. However, a robust response did not occur in a few patients, and infections in such patients may be undetectable by serologic and seroepidemiologic methods.

Technical Appendix Demographic and clinical profiles for patients and serologic and statistical methods in a study of the kinetics of serologic responses to MERS-CoV infection in humans.
