# Supplementary material for: Kinetics of Serologic Responses to MERS Coronavirus Infection in Humans, South Korea
Source: Emerg Infect Dis. 2015 Dec;21(12):2186–9. doi: 10.3201/eid2112.151421 (PMC4672454; doi:10.3201/eid2112.151421)
Supplement: Technical Appendix — Demographic and clinical profiles for patients and serologic and statistical methods in a study of the kinetics of serologic responses to MERS-CoV infection in humans. [file 15-1421-Techapp-s1.pdf]

Article DOI: http://dx.doi.org/10.3201/eid2112.151421

Kinetics of Serologic Responses to MERS Coronavirus Infection in Humans, South Korea

Technical Appendix

Methods

Serologic Tests

The serum samples were heat-inactivated for 30 minutes at 56°C before testing. Sequential serum samples from the patients were analyzed for MERS-CoV antibody by plaque reduction neutralization tests (PRNT) and IgG ELISA tests. Sequential serum samples from each patient were tested in parallel.

The PRNTs were performed in a 24-well format in duplicate for each serum dilution. Two-fold serum dilutions (starting dilution of 1:10) were incubated with 40–60 PFUs of virus for 1 h at 37°C. The virus–serum mixture was added onto the Vero cells monolayer for 1hr at 37°C in a 5% CO incubator. Then, the supernatant was removed and the cells overlaid with 1% Agarose (SeaKem LE Agarose; Lonza, Switzerland) in cell culture medium (Minimum Essential Medium with 2% fetal bovine serum). After 3 days, the plates were fixed and stained. The titers were determined by defining the highest serum dilutions that resulted in ≥50% ($PRNT_{50}$) reduction in the number of plaques (*1*,*2*). Positive and negative controls and a virus back-titration were included in each assay.

The S1 ELISA EI 2604–9601G kit was purchased from EUROIMMUN AGfor detection of human IgG against MERS-CoV (http://www.euroimmun.com) and the test was done according to the manufacturer’s instructions (*1*). The assay includes a calibrator which defines the upper limit of the reference range in non-infected humans and this value is defined as the cut off. The assay is made semiquantitative by calculating the ratio of the extinction of the patient

sample/ extinction of the calibrator. Ratios <0.8 is considered negative, those ≥1.1 as positive and those ≥0.8 to <1.1 regarded as borderline.

Statistical Methods

We fitted accelerated failure time models assuming a lognormal distribution to compare time from illness onset to the log phase of antibody response measured by $PRNT_{50}$ and ELISA optical density (OD) ratios, accounting for interval censoring due to time of testing. The model was also used to identify factors associated with longer time to the log phase of antibody response, including disease severity, and other factors such as sex, age, incubation period, use of steroid and antivirals and comorbid conditions adjusted for disease severity. The model can be specified as

$$Y = \log(T) = \mu + \beta' X + \sigma\varepsilon$$

where T is the duration from illness onset to commencement of antibody response, X are the factors of interest, β and σ are the intercept and scale parameters and ε is the error term. Similar analyses were conducted to compare time from illness onset to $PRNT_{50}$ titers reaching 1:40 and ELISA positive (OD ratios ≥1.1), respectively. The anti-log of the estimated coefficient β for the factor of interest is presented as the acceleration factor, which is interpreted as the multiplier on the median time length from illness onset to the commencement of different antibody responses.

We also identified any of the above factors which associated with a steeper rate of increase in $PRNT_{50}$ titers and ELISA OD ratios during the log phase, adjusted for disease severity. We visually excluded data in the lag and steady-state phase and fitted linear mixed models assuming a first-order autoregressive structure to account for repeated measurements, assuming a linear increasing trend by days since illness onset.

$$Y_{ij} = \beta' X_i + b_i T_{ij} + \varepsilon$$

where Y_{ij} is the jth measurement for patient i on day T_{ij} since illness onset, X_i are the above factors of interest including days since illness onset and ε is the error term. b_i is assumed to follow a multivariate normal distribution with first-order autoregressive structure, i.e., covariances $\gamma_{ts} \propto \rho^{|t-s|}$. The estimated coefficients of the interaction term between the above factors and days since illness onset indicate the potential differences in the rate of increase in $PRNT_{50}$ titers and ELISA OD ratios. For analyses based on continuous measurements, titers were first log-transformed

(with base 10). All statistical tests were considered significant at the level of $p<0.05$ and were conducted by using R version 3.1.2 (https://www.r-project.org/).

References

1. Meyer B, Drosten C, Müller MA. Serological assays for emerging coronaviruses: challenges and pitfalls. Virus Res. 2014;194:175–83. PubMed http://dx.doi.org/10.1016/j.virusres.2014.03.018

2. Muth D, Corman VM, Meyer B, Assiri A, Al-Masri M, Farah M, et al. Infectious Middle East respiratory syndrome coronavirus excretion and serotype variability based on live virus isolates from patients in Saudi Arabia. J Clin Microbiol. 2015;53:2951–5. Epub 2015 Jul 8. PubMed http://dx.doi.org/10.1128/JCM.01368-15

Technical Appendix Table 1. Demographic and clinical profiles of patients with Middle East respiratory syndrome coronavirus infection

Patient	Sex/age, y	Underlying disease	Oxygen therapy	Mechanical ventilation	Corticosteroid use	Antiviral drug use†	Outcome
A	M/38		Yes	Yes	Yes	Yes	Hospitalized (as of D77)‡
B	M/65		Yes	Yes	Yes	Yes	Hospitalized (as of D70)‡
C	M/55		Yes	Yes	No	Yes	Discharged
D	M/35	Pneumonia	Yes	Yes	Yes	Yes	Discharged
E	F/79	CHD, CKD, dementia Bladder cancer	Yes	Yes§	Yes	Yes	Died
F	M/55	DM, CPD, lung abscess	Yes	No	Yes	No	Discharged
G	M/56		Yes	No	No	Yes	Discharged
H	M/71	DM, CVA	Yes	No	No	No	Discharged
I	F/77	DM, asthma	Yes	No	No	No	Discharged
J	M/76	DM, CHD, dementia	No	No	No	No	Discharged
K	M/59	CHD	No	No	No	Yes	Discharged
L	F/56		No	No	No	No	Discharged
M	M/56	DM, CHD, CLD, pulmonary tuberculosis	No	No	No	No	Discharged
N	F/54		No	No	No	No	Discharged
O	M/46		No	No	No	No	Discharged
P	M/35		No	No	No	Yes	Discharged
Q	M/52	Liver abscess	No	No	No	Yes	Discharged

*Gray shading indicates patients with severe disease. CHD, chronic heart failure; CKD, chronic kidney disease; CLD, chronic liver disease; CPD, chronic pulmonary disease; CVA, cerebrovascular accident; DM, diabetes mellitus.
†Interferon and ribavirin +/– lopinavir/ritonavir.
‡Patient status on August 13, 2015.
§Noninvasive mechanical ventilator.

Technical Appendix Table 2. Association and p values for different clinical factors with time from illness onset to $PRNT_{50}$ titers reaching 1:40 and S1-ELISA antibody reaching positive cut off value*

	Acceleration factor of time from illness onset to reaching respective antibody level			
Clinical factors	$PRNT_{50}$ titer ≥1:40	p value	S1-ELISA positive	p value
Severe disease	1.03	0.89	0.91	0.65
Male sex†	0.76	0.24	0.88	0.54
Age ≥60 y†	1.07	0.78	0.94	0.77
Incubation period†	0.96	0.14	0.92	<001
Use of corticosteroid†	1.23	0.51	1.07	0.78
Use of antiviral drugs†	0.76	0.19	0.84	0.37
Concomitant conditions†	0.94	0.79	0.93	0.72

*Accelerated failure time models were used; acceleration factor >1 means a longer interval to reaching the threshold. $PRNT_{50}$, 50% endpoint plaque reduction neutralization test.
†Effects were adjusted for severity.
